# Supplementary material for: Dynamic representation of 3D auditory space in the midbrain of the free-flying echolocating bat
Source: eLife. 2018 Apr 10;7:e29053. doi: 10.7554/eLife.29053 (PMC5896882; doi:10.7554/eLife.29053)
Supplement: Supplementary file 1. — The SSG and non-SSG distance tuning distributions were compared using the non-parametric Brown-Forsythe Test at the level α of 0.05. Cells in red show a significant sharpening in the distance tuning distribution when the bat emitted SSGs as compared to the variance of the distance tuning distribution when the bat produced single calls (non-SSGs). Cells in gray did not show a significant effect. Cells in blue showed a significant effect but in the opposite direction. (B) Comparison of the SSG and non-SSG distance tuning distributions for each cell in Figure 5F. The SSG and non-SSG distance tuning distributions were compared using the non-parametric Wilcoxon Rank Sum Test at an α of 0.05. Cells marked with red ink show a significant shortening in distance tuning for SSGs as compared to the condition when the bat produces single calls (non-SSGs). Cells marked with gray ink did not show a significant effect. [file elife-29053-supp1.docx]

**Supplementary File**

**Supplementary File 1A. Comparison of the variance of SSG and non-SSG distance tuning distributions for each cell in Fig. 4d**. The SSG and non-SSG distance tuning distributions were compared using the non-parametric Brown-Forsythe Test at the level α of 0.05. Cells in red show a significant sharpening in the distance tuning distribution when the bat emitted SSGs as compared to the variance of the distance tuning distribution when the bat produced single calls (non-SSGs). Cells in gray did not show a significant effect. Cells in blue showed a significant effect but in the opposite direction.

| **No** | **Bat ID** | **Statistic** | **df** | **p-value** | **Permutation test (p-value)** |
| --- | --- | --- | --- | --- | --- |
| 1 | 1 | 0.8127 | 41 | 0.158 | 0.073 |
| 2 | 1 | 1.5934 | 31 | 0.0854 | 0.102 |
| 3 | 1 | 19.7105 | 60 | < 10^-3^ | < 10^-3^ |
| 4 | 1 | 13.8958 | 48 | < 10^-4^ | < 10^-3^ |
| 5 | 1 | 72.2865 | 153 | < 10^-4^ | < 10^-3^ |
| 6 | 1 | 18.6761 | 87 | < 10^-4^ | < 10^-3^ |
| 7 | 1 | 71.203 | 158 | < 10^-4^ | < 10^-3^ |
| 8 | 1 | 10.6497 | 47 | < 10^-3^ | < 10^-3^ |
| 9 | 1 | 31.1736 | 60 | < 10^-3^ | < 10^-3^ |
| 10 | 1 | 13.53 | 29 | < 10^-3^ | < 10^-3^ |
| 11 | 1 | 0.8127 | 124 | 0.0254 | 0.002 |
| 12 | 1 | 7.4073 | 35 | 0.2357 | 0.291 |
| 13 | 1 | 2.8241 | 84 | 0.0649 | 0.019 |
| 14 | 1 | 2.8438 | 35 | 0.4363 | 0.658 |
| 15 | 1 | 0.1821 | 67 | 0.0019 | 0.001 |
| 16 | 1 | 6.2021 | 52 | 0.7348 | 0.781 |
| 17 | 1 | 0.0342 | 37 | 0.6574 | 0.768 |
| 18 | 1 | 0.0006 | 55 | 0.4187 | 0.365 |
| 19 | 1 | 0.3857 | 45 | < 10^-3^ | < 10^-3^ |
| 20 | 1 | 12.6318 | 58 | 0.2887 | 0.289 |
| 21 | 1 | 2.3855 | 77 | < 10^-3^ | < 10^-3^ |
| 22 | 1 | 14.2166 | 36 | 0.001 | 0.003 |
| 23 | 1 | 8.8835 | 33 | 0.4076 | 0.389 |
| 24 | 1 | 0.6151 | 56 | 0.1683 | 0.296 |
| 25 | 1 | 2.5335 | 30 | 0.0037 | 0.007 |
| 26 | 1 | 9.7524 | 52 | 0.0821 | 0.006 |
| 27 | 1 | 2.2625 | 37 | 0.559 | 0.165 |
| 28 | 2 | 6.3734 | 55 | 0.003 | 0.002 |
| 29 | 2 | 1.8511 | 49 | 0.0425 | 0.039 |
| 30 | 2 | 4.2135 | 34 | 0.9881 | 0.912 |
| 31 | 2 | 0.6418 | 56 | 0.1781 | 0.335 |
| 32 | 2 | 0.2039 | 31 | 0.5679 | 0.641 |
| 33 | 2 | 0.0937 | 67 | 0.4728 | 0.757 |
| 34 | 2 | 0.2947 | 34 | 0.6719 | 0.598 |
| 35 | 2 | 0.1328 | 70 | 0.4914 | 0.682 |
| 36 | 2 | 9.0981 | 48 | < 10^-4^ | 0.004 |
| 37 | 2 | 0.0001 | 41 | 0.3731 | 0.991 |
| 38 | 2 | 0.0006 | 33 | 0.4174 | 0.977 |
| 39 | 2 | 4.1244 | 29 | 0.8988 | 0.738 |
| 40 | 2 | 1.9103 | 34 | 0.7589 | 0.912 |
| 41 | 2 | 0.4294 | 35 | 0.3746 | 0.461 |
| 42 | 2 | 1.7645 | 37 | 0.0656 | 0.044 |
| 43 | 2 | 3.1353 | 35 | < 10^-3^ | 0.058 |
| 44 | 2 | 2.5841 | 67 | 0.0625 | 0.011 |
| 45 | 2 | 0.9236 | 40 | 0.0949 | 0.043 |
| 46 | 2 | 6.7482 | 84 | 0.014 | 0.006 |
| 47 | 2 | 5.0299 | 33 | 0.9554 | 0.899 |
| 48 | 2 | 4.0942 | 68 | 0.0234 | 0.036 |
| 49 | 2 | 8.5828 | 108 | 0.0074 | 0.001 |
| 50 | 2 | 2.2051 | 77 | 0.0388 | < 10^-3^ |
| 51 | 2 | 8.5589 | 115 | < 10^-4^ | < 10^-4^ |
| 52 | 2 | 5.0477 | 38 | 0.0497 | 0.029 |
| 53 | 2 | 4.6178 | 128 | 0.028 | 0.024 |

**Supplementary File 1B. Comparison of the SSG and non-SSG distance tuning distributions for each cell in Fig. 4f.** The SSG and non-SSG distance tuning distributions were compared using the non-parametric Wilcoxon Rank Sum Test at an α of 0.05. Cells marked with red ink show a significant shortening in distance tuning for SSGs as compared to the condition when the bat produces single calls (non-SSGs). Cells marked with gray ink did not show a significant effect.

| **No** | **Bat ID** | **Wilcoxon rank-sum**  **test p-value at α = 0.05** | **Permutation test (p-value)** |
| --- | --- | --- | --- |
| 1 | 1 | 0.01 | 0.003 |
| 2 | 1 | 0.06 | 0.182 |
| 3 | 1 | < 10^-4^ | 0.061 |
| 4 | 1 | < 10^-3^ | 0.001 |
| 5 | 1 | < 10^-3^ | < 10^-3^ |
| 6 | 1 | < 10^-4^ | < 10^-3^ |
| 7 | 1 | < 10^-3^ | 0.002 |
| 8 | 1 | < 10^-3^ | 0.001 |
| 9 | 1 | 0.01 | 0.009 |
| 10 | 1 | < 10^-4^ | 0.007 |
| 11 | 1 | 0.21 | 0.361 |
| 12 | 1 | < 10^-3^ | < 10^-3^ |
| 13 | 1 | 0.32 | 0.491 |
| 14 | 1 | 0.29 | 0.312 |
| 15 | 1 | < 10^-3^ | 0.001 |
| 16 | 1 | 0.15 | 0.215 |
| 17 | 1 | < 10^-3^ | < 10^-3^ |
| 18 | 1 | < 10^-4^ | 0.004 |
| 19 | 1 | < 10^-3^ | 0.007 |
| 20 | 1 | < 10^-3^ | 0.002 |
| 21 | 1 | 0.18 | 0.001 |
| 22 | 1 | < 10^-4^ | < 10^-3^ |
| 23 | 1 | < 10^-3^ | < 10^-3^ |
| 24 | 1 | 0.01 | 0.01 |
| 25 | 2 | < 10^-3^ | < 10^-3^ |
| 26 | 2 | 0.03 | 0.01 |
| 27 | 2 | 0.34 | 0.728 |
| 28 | 2 | 0.01 | < 10^-3^ |
| 29 | 2 | 0.2 | 0.633 |
| 30 | 2 | < 10^-3^ | < 10^-3^ |
| 31 | 2 | < 10^-4^ | 0.069 |
| 32 | 2 | 0.01 | 0.014 |
| 33 | 2 | 0.01 | 0.002 |
| 34 | 2 | < 10^-3^ | 0.005 |
| 35 | 2 | 0.75 | 0.869 |
| 36 | 2 | 0.63 | 0.589 |
| 37 | 2 | 0.88 | 0.822 |
| 38 | 2 | 0.96 | 0.983 |
| 39 | 2 | 0.34 | 0.361 |
| 40 | 2 | 0.46 | 0.322 |
| 41 | 2 | 0.12 | 0.448 |
| 42 | 2 | 0.07 | 0.124 |
| 43 | 2 | < 10^-3^ | 0.065 |
| 44 | 2 | < 10^-3^ | 0.061 |
| 45 | 2 | 0.02 | < 10^-3^ |
| 46 | 2 | 0.06 | 0.021 |
| 47 | 2 | 0.01 | 0.039 |
| 48 | 2 | 0.05 | 0.012 |
| 49 | 2 | 0.26 | 0.435 |
| 50 | 2 | < 10^-4^ | < 10^-3^ |
| 51 | 2 | < 10^-4^ | 0.012 |
