# Supplementary material for: Dynamic representation of 3D auditory space in the midbrain of the free-flying echolocating bat
Source: eLife. 2018 Apr 10;7:e29053. doi: 10.7554/eLife.29053 (PMC5896882; doi:10.7554/eLife.29053)
Supplement: Transparent reporting form [file elife-29053-transrepform.pdf]

eLIFE
1st Floor
24 Hills Road
Cambridge CB2 1JP, UK
P 01223 855340
W elifesciences.org
T @elife

# *eLife's* transparent reporting form

We encourage authors to provide detailed information *within their submission* to facilitate the interpretation and replication of experiments. If you have any questions, please contact us: editorial@elifesciences.org.

## Sample-size estimation

- You should state whether an appropriate sample size was computed when the study was being designed
- You should state the statistical method of sample size computation and any required assumptions
- If no explicit power analysis was used, you should describe how you decided what sample (replicate) size (number) to use

Please outline where this information can be found within the submission (e.g., page numbers or figure legends), or explain why this information doesn't apply to your submission:

1) This study is based on behaving, free-flying bats engaged in a naturalistic task, where the bat's flight pattern, number of calls/echoes are under control of the experimental animal and outside the control of the experimenter. Due to this aspect of the experiment, the traditional method of explicit power analysis was not possible.
2) Information regarding the number of trials, session, calls and echoes evoked and used for computing 3D receptive fields is described in the following sections – Main article pages 6 & 7, Methods section – pages 25 & 31-33 as well as displayed graphically in Figure 3-figure supplement 1, Figure legend – page 43.
3) Details of number of neurons, spike sorting and selection criterion are described in detail main text – pages 2 & 6, Methods section – pages 24 & 25.
4) Details of selection criterion for single neurons for the analysis which tests the sharpness and peak tuning of spatial tuning (described in Figure 5) when bats produce SSGs v/s nonSSG is described in the main text – page 7-8, Figure 5, Methods – page 34

## Replicates

- You should report how often each experiment was performed
- You should include a definition of biological versus technical replication
- The data obtained should be provided and sufficient information should be provided to indicate the number of independent biological and/or technical replicates
- If you encountered any outliers, you should describe how these were handled

- Criteria for exclusion/inclusion of data should be clearly stated
- High-throughput sequence data should be uploaded before submission, with a private link for reviewers provided (these are available from both GEO and ArrayExpress)

Please outline where this information can be found within the submission (e.g., page numbers or figure legends), or explain why this information doesn't apply to your submission:

1) The details of the experiments are provided the main text – pages 3 & 4, Methods section – pages 22-26.
2) Information regarding the number of trials, session, calls and echoes evoked and used for computing 3D receptive fields is described in the following sections – Main article pages 6 & 7, Methods section – pages 25 & 31-33 as well as displayed graphically in Figure 3-figure supplement 1, Figure legend – page 43.
3) Details of the construction and validation of the *echo model* are provided in the main text (page 4-6, Figure 2 , Figure 2-figure supplement 1 and 2 – legends – page 42, Methods section – pages 26-31)
4) Details of number of neurons, spike sorting and selection criterion are described in detail main text – pages 2 & 6, Methods section – pages 24 & 25.
5) Criteria for identifying Sonar Sound Groups is provided in Figure 6 as well as in the Methods section – page #26.
6) Details of selection criterion for single neurons for the analysis which tests the sharpness and peak tuning of spatial tuning (described in Figure 5) when bats produce SSGs v/s nonSSG is described in the main text – page 7-8, Figure 5, Methods – page 34

**Statistical reporting**

- Statistical analysis methods should be described and justified
- Raw data should be presented in figures whenever informative to do so (typically when N per group is less than 10)
- For each experiment, you should identify the statistical tests used, exact values of N, definitions of center, methods of multiple test correction, and dispersion and precision measures (e.g., mean, median, SD, SEM, confidence intervals; and, for the major substantive results, a measure of effect size (e.g., Pearson's r, Cohen's d)
- Report exact p-values wherever possible alongside the summary statistics and 95% confidence intervals. These should be reported for all key questions and not only when the p-value is less than 0.05.

Please outline where this information can be found within the submission (e.g., page numbers or figure legends), or explain why this information doesn't apply to your submission:

1) Mathematical proofs explaining the construction and validation of the *echo model* are provided in the main text (page 5, Figure 2, Figure 2-figure supplement 1 and 2 – legends – page 42, Methods section – pages 26-31)
2) Raw neural data is shown in Figure 1 as well as in Figure 1-figure supplement 1 – legend – page 42. Figure 3 also shows raw neural data which is used to build spatial receptive fields.
3) Raw acoustic data is shown in Figure 1.
4) Raw data demonstrating the construction of 3D spatial receptive fields is shown in Figure 3.
5) Raw flight data is shown in Figure 1, Figure 2 and in Figure 5.
6) Statistical tests and N values for Figure 5 () are provided in the main text on page 8. Details of the statistical test performed on each cell with F value's and p-values is provided in Supplementary Files 1A and 1B – legends – page 45

(For large datasets, or papers with a very large number of statistical tests, you may upload a single table file with tests, Ns, etc., with reference to page numbers in the manuscript.)

**Additional data files ("source data")**

- We encourage you to upload relevant additional data files, such as numerical data that are represented as a graph in a figure, or as a summary table
- Where provided, these should be in the most useful format, and they can be uploaded as "Source data" files linked to a main figure or table
- Include model definition files including the full list of parameters used
- Include code used for data analysis (e.g., R, MatLab)
- Avoid stating that data files are "available upon request"

Please indicate the figures or tables for which source data files have been provided:

1) Summary tables for data are provided in Supplementary Files 1A and 1B.
2) Matlab code as well as raw data for reconstructing each figure with statistical tests will be uploaded at a later stage.
